# 1,3-Dibenzyl-1*H*-anthra[1,2-*d*]imidazole-2,6,11(3*H*)-trione

**DOI:** 10.1107/S1600536811015078

**Published:** 2011-04-29

**Authors:** Zahra Afrakssou, Youssef Kandri Rodi, Frédéric Capet, El Mokhtar Essassi, Lahcen El Ammari

**Affiliations:** a Laboratoire de Chimie Organique Appliquée, Université Sidi Mohamed Ben Abdallah, Faculté des Sciences et Techniques, Route d’Immouzzer, BP 2202 Fès, Morocco; b Unité de Catalyse et de Chimie du Solide (UCCS), UMR 8181, Ecole Nationale Supérieure de Chimie de Lille, France; c Laboratoire de Chimie Organique Hétérocyclique URAC21, Faculté des Sciences, Université Mohammed V-Agdal, Avenue Ibn Battouta, BP 1014, Rabat, Morocco; d Laboratoire de Chimie du Solide Appliquée, Faculté des Sciences, Université Mohammed V-Agdal, Avenue Ibn Battouta, BP 1014, Rabat, Morocco

## Abstract

The mol­ecule of the title compound, C_29_H_20_N_2_O_3_, contains four fused rings, three are six-membered rings and one is the five-membered imidazole ring. The fused-ring system is linked to two benzyl groups. The four fused rings are folded around the O=C⋯C=O direction of the anthraquinone, with a dihedral angle of 16.36 (8)° between the two terminal rings (*A* and *D*). The imidazole ring (*D*) is almost perpendicular to the two benzyl groups (*E* and *F*) with dihedral angles of 86.69 (17) and 83.15 (13)°, respectively. In the crystal, adjacent mol­ecules are linked by inter­molecular C—H⋯O hydrogen bonding.

## Related literature

For background to the pharmacological activity of anthraquinone, see: Alves *et al.* (2004 ▶); Gatto *et al.* (1996 ▶); Krapcho *et al.* (1991 ▶). For information on its use as a synthetic dye, see: Naeimi & Namdari (2009 ▶). For related structures, see: Afrakssou *et al.* (2010 ▶); Guimarães *et al.* (2009 ▶). For puckering parameters, see: Cremer & Pople (1975 ▶).
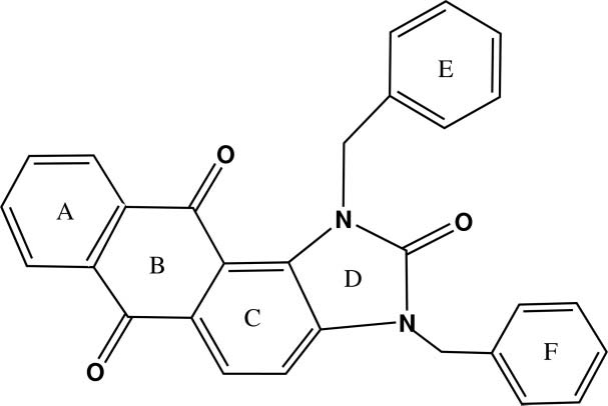


## Experimental

### 

#### Crystal data

C_29_H_20_N_2_O_3_
*M* *_r_* = 444.47
Orthorhombic, 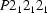

*a* = 8.1389 (3) Å
*b* = 12.8748 (4) Å
*c* = 21.5528 (8) Å
*V* = 2258.45 (14) Å^3^
*Z* = 4
Mo *K*α radiation
μ = 0.09 mm^−1^
*T* = 296 K
0.49 × 0.18 × 0.15 mm

#### Data collection

Bruker APEXII CCD diffractometer
Absorption correction: multi-scan (*SADABS*; Bruker, 2009 ▶) *T*_min_ = 0.982, *T*_max_ = 0.987
36228 measured reflections
2629 independent reflections
2137 reflections with *I* > 2σ(*I*)
*R*_int_ = 0.047

#### Refinement

*R*[*F* ^2^ > 2σ(*F* ^2^)] = 0.039
*wR*(*F* ^2^) = 0.108
*S* = 1.05
2629 reflections
308 parameters
H-atom parameters constrained
Δρ_max_ = 0.23 e Å^−3^
Δρ_min_ = −0.18 e Å^−3^

### 

Data collection: *APEX2* (Bruker, 2009 ▶); cell refinement: *SAINT-Plus* (Bruker, 2009 ▶); data reduction: *SAINT-Plus*; program(s) used to solve structure: *SHELXS97* (Sheldrick, 2008 ▶); program(s) used to refine structure: *SHELXL97* (Sheldrick, 2008 ▶); molecular graphics: *ORTEPIII* (Burnett & Johnson, 1996 ▶) and *ORTEP-3 for Windows* (Farrugia, 1997 ▶); software used to prepare material for publication: *SHELXL97*.

## Supplementary Material

Crystal structure: contains datablocks I, global. DOI: 10.1107/S1600536811015078/dn2679sup1.cif

Structure factors: contains datablocks I. DOI: 10.1107/S1600536811015078/dn2679Isup2.hkl

Supplementary material file. DOI: 10.1107/S1600536811015078/dn2679Isup3.cml

Additional supplementary materials:  crystallographic information; 3D view; checkCIF report

## Figures and Tables

**Table 1:** Hydrogen-bond geometry (Å, °)

*D*—H⋯*A*	*D*—H	H⋯*A*	*D*⋯*A*	*D*—H⋯*A*
C13—H13⋯O1^i^	0.93	2.39	3.312 (3)	171
C23—H23*A*⋯O2^ii^	0.97	2.47	3.439 (4)	174

Symmetry codes: (i) 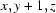; (ii) 
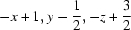
.

**Fig. 1.**
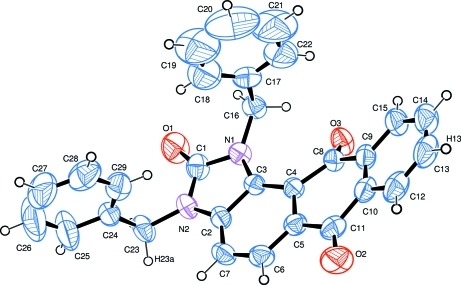
Molecular plot the title compound with the atom-labelling scheme. Displacement ellipsoids are drawn at the 50% probability level. H atoms are represented as small circles.

## supplementary crystallographic information

### Comment

Anthraquinone-containing extracts from different plant sources such as senna, cascara, aloe, frangula, and rhubarb have been found to have wide variety of pharmacological activities such as antiinflammatory, wound healing, analgesic, antipyretic, antimicrobial, and antitumor activities (Alves *et al.*, 2004). Anthraquinone planarity allows an intercalation between base pairs of DNA in the β conformation, while its redox properties are linked to the production of radical species in biological systems. The chemical and biological activity of anthraquinone compounds depends on the different substituents of the planar ring system (Gatto *et al.*, 1996; Krapcho *et al.*, 1991). Anthraquinone dyes are used for coloration of cotton and cellulose fibers as well as of hydrophobic, synthetic materials (Naeimi *et al.*, 2009).

The present work is a continuation of the preparation of new derivatives of anthra[1,2-*d*]imidazole-2,6,11–trione for biological applications (Afrakssou *et al.*, (2010), Guimarães *et al.* (2009)). The reactivity of benzyl bromide towards 1*H*-anthra [2,1-*d*]imidazole-2,6,11(3*H*)-trione under phase-transfer catalysis conditions using tetra *n*-butyl ammonium bromide (TBAB) as catalyst and potassium carbonate as base leads to 1,3-dibenzyl-1*H*-anthra[2,1-*d*]imidazole-2,6,11(3*H*)-trione with good yield (Scheme 1).

All rings forming the molecule are planar except for the anthraquinone (B) which adopts a twisted conformation (Fig. 1), as indicated by Cremer & Pople (1975) with puckering parameters Q = 0.242 (2) Å, θ = 104.7 (5) ° and φ = 137.6 (5)°. The fused five and six-membered rings (C, D) are planar and built with A ring (sheme 1) a dihedral angle of 16.36 (8) °. The imidazole ring (D) is almost perpendicular to the two benzyl groups (E, F) with dihedral angles of 86.69 (17) ° and 83.15 (13) ° respectively. In the crystal, adjacent molecules are linked by intermolecular C—H···O hydrogen bonding (Table 1). The structure is further stabilized by π-π interactions between rings A and D with a centroid to centroid distance of 3.411 (2) Å and an interplanar distance  of 3.395 (2)Å resulting in slight offset of 5.32°.

### Experimental

To a solution of 1*H*-anthra [2, 1 - d] imidazole-2, 6, 11(3*H*)-trione (0.5 g, 0.18 mmol), potassium carbonate (0.78 g, 0.56 mmol) and tetra n-butylammonium bromide (0.06 g, 0.018 mmol) in DMF (15 ml)) was added Benzyl bromide (0.56 ml, 0.47 mmol). Stirring was continued at room temperature for 24 h. The mixture was filtered and the solvent removed. The residue was extracted with water. The organic compound was chromatographed on a column of silica gel with ethyl acetate-hexane (1/1) as eluent. Orange crystals were isolated when the solvent was allowed to evaporate.

### Refinement

H atoms were located in a difference map and treated as riding with C—H = 0.93 Å for all H atoms with *U*_iso_(H) = 1.2 *U*_eq_ aromatic and methylene. In the absence of significant anomalous scattering, the absolute structure could not be reliably determined and thus the Friedel pairs were merged and any references to the Flack parameter were removed. The reflections (0 0 2) and (0 1 1) were omitted because the difference between their calculated and observed intensities are very large. They are affected by the beamstop.

### Crystal data

**Table:**

C_29_H_20_N_2_O_3_	*F*(000) = 928
*M**_r_* = 444.47	*D*_x_ = 1.307 Mg m^−^^3^
Orthorhombic, *P*2_1_2_1_2_1_	Mo *K*α radiation, λ = 0.71073 Å
Hall symbol: P 2ac 2ab	Cell parameters from 2629 reflections
*a* = 8.1389 (3) Å	θ = 2.5–26.4°
*b* = 12.8748 (4) Å	µ = 0.09 mm^−^^1^
*c* = 21.5528 (8) Å	*T* = 296 K
*V* = 2258.45 (14) Å^3^	Flat, orange
*Z* = 4	0.49 × 0.18 × 0.15 mm

### Data collection

**Table:**

Bruker APEXII CCD diffractometer	2629 independent reflections
Radiation source: fine-focus sealed tube	2137 reflections with *I* > 2σ(*I*)
graphite	*R*_int_ = 0.047
φ and ω scans	θ_max_ = 26.4°, θ_min_ = 2.5°
Absorption correction: multi-scan (*SADABS*; Bruker, 2009)	*h* = −10→10
*T*_min_ = 0.982, *T*_max_ = 0.987	*k* = −15→16
36228 measured reflections	*l* = −26→26

### Refinement

**Table:**

Refinement on *F*^2^	Secondary atom site location: difference Fourier map
Least-squares matrix: full	Hydrogen site location: inferred from neighbouring sites
*R*[*F*^2^ > 2σ(*F*^2^)] = 0.039	H-atom parameters constrained
*wR*(*F*^2^) = 0.108	*w* = 1/[σ^2^(*F*_o_^2^) + (0.0603*P*)^2^ + 0.2421*P*] where *P* = (*F*_o_^2^ + 2*F*_c_^2^)/3
*S* = 1.05	(Δ/σ)_max_ < 0.001
2629 reflections	Δρ_max_ = 0.23 e Å^−^^3^
308 parameters	Δρ_min_ = −0.18 e Å^−^^3^
0 restraints	Extinction correction: *SHELXL97* (Sheldrick, 2008), Fc^*^=kFc[1+0.001xFc^2^λ^3^/sin(2θ)]^-1/4^
Primary atom site location: structure-invariant direct methods	Extinction coefficient: 0.0123 (15)

### Special details

**Table:**

Geometry. All s.u.'s (except the s.u. in the dihedral angle between two l.s. planes) are estimated using the full covariance matrix. The cell s.u.'s are taken into account individually in the estimation of s.u.'s in distances, angles and torsion angles; correlations between s.u.'s in cell parameters are only used when they are defined by crystal symmetry. An approximate (isotropic) treatment of cell s.u.'s is used for estimating s.u.'s involving l.s. planes.
Refinement. Refinement of *F*^2^ against ALL reflections. The weighted *R*-factor *wR* and goodness of fit *S* are based on *F*^2^, conventional *R*-factors *R* are based on *F*, with *F* set to zero for negative *F*^2^. The threshold expression of *F*^2^ > σ(*F*^2^) is used only for calculating *R*-factors(gt) *etc*. and is not relevant to the choice of reflections for refinement. *R*-factors based on *F*^2^ are statistically about twice as large as those based on *F*, and *R*- factors based on ALL data will be even larger.

### Fractional atomic coordinates and isotropic or equivalent isotropic displacement parameters (Å^2^)

**Table:**

	*x*	*y*	*z*	*U*_iso_*/*U*_eq_	
C1	0.0570 (3)	0.41604 (19)	0.84012 (11)	0.0512 (6)	
C2	0.2441 (3)	0.53761 (17)	0.81399 (10)	0.0420 (5)	
C3	0.0919 (3)	0.58851 (17)	0.82177 (9)	0.0375 (5)	
C4	0.0807 (3)	0.69572 (17)	0.81222 (9)	0.0387 (5)	
C5	0.2294 (3)	0.74849 (19)	0.79858 (10)	0.0439 (5)	
C6	0.3764 (3)	0.6961 (2)	0.79119 (12)	0.0508 (6)	
H6	0.4711	0.7333	0.7818	0.061*	
C7	0.3851 (3)	0.5888 (2)	0.79761 (11)	0.0503 (6)	
H7	0.4829	0.5532	0.7910	0.060*	
C8	−0.0748 (3)	0.75336 (18)	0.80889 (10)	0.0425 (5)	
C9	−0.0669 (3)	0.86723 (18)	0.81962 (10)	0.0471 (6)	
C10	0.0803 (3)	0.92029 (19)	0.81274 (10)	0.0507 (6)	
C11	0.2320 (4)	0.8639 (2)	0.79481 (12)	0.0546 (6)	
C12	0.0849 (5)	1.0278 (2)	0.82132 (12)	0.0674 (8)	
H12	0.1826	1.0642	0.8158	0.081*	
C13	−0.0569 (5)	1.0794 (2)	0.83799 (13)	0.0758 (9)	
H13	−0.0538	1.1507	0.8449	0.091*	
C14	−0.2029 (5)	1.0266 (2)	0.84455 (13)	0.0730 (9)	
H14	−0.2975	1.0624	0.8558	0.088*	
C15	−0.2100 (4)	0.9207 (2)	0.83451 (13)	0.0606 (7)	
H15	−0.3095	0.8855	0.8377	0.073*	
C16	−0.1814 (3)	0.5233 (2)	0.86850 (12)	0.0506 (6)	
H16A	−0.2310	0.4552	0.8728	0.061*	
H16B	−0.2530	0.5651	0.8426	0.061*	
C17	−0.1677 (3)	0.5729 (2)	0.93142 (12)	0.0590 (7)	
C18	−0.0688 (5)	0.5290 (4)	0.97586 (16)	0.1043 (13)	
H18	−0.0091	0.4694	0.9667	0.125*	
C19	−0.0579 (8)	0.5726 (6)	1.0333 (2)	0.155 (2)	
H19	0.0095	0.5425	1.0631	0.186*	
C20	−0.1443 (10)	0.6593 (6)	1.0473 (2)	0.164 (3)	
H20	−0.1352	0.6888	1.0866	0.197*	
C21	−0.2439 (9)	0.7032 (4)	1.0045 (3)	0.152 (3)	
H21	−0.3042	0.7624	1.0140	0.183*	
C22	−0.2544 (6)	0.6587 (3)	0.94659 (17)	0.1007 (13)	
H22	−0.3230	0.6886	0.9171	0.121*	
C23	0.3437 (3)	0.3524 (2)	0.82695 (13)	0.0583 (7)	
H23A	0.4241	0.3652	0.7946	0.070*	
H23B	0.2925	0.2859	0.8184	0.070*	
C24	0.4309 (3)	0.34610 (19)	0.88834 (12)	0.0522 (6)	
C25	0.5405 (5)	0.2681 (3)	0.8964 (2)	0.1062 (14)	
H25	0.5582	0.2207	0.8645	0.127*	
C26	0.6266 (7)	0.2583 (5)	0.9517 (3)	0.143 (2)	
H26	0.7019	0.2045	0.9563	0.172*	
C27	0.6028 (6)	0.3250 (4)	0.9984 (2)	0.1106 (15)	
H27	0.6577	0.3162	1.0359	0.133*	
C28	0.4987 (5)	0.4050 (3)	0.99050 (14)	0.0869 (10)	
H28	0.4856	0.4536	1.0221	0.104*	
C29	0.4106 (4)	0.4159 (2)	0.93574 (13)	0.0680 (8)	
H29	0.3374	0.4709	0.9312	0.082*	
N1	−0.0211 (2)	0.51260 (15)	0.83808 (8)	0.0440 (5)	
N2	0.2183 (2)	0.43334 (15)	0.82446 (9)	0.0484 (5)	
O1	−0.0047 (3)	0.33329 (14)	0.85284 (11)	0.0743 (6)	
O2	0.3554 (3)	0.91046 (17)	0.77953 (12)	0.0851 (7)	
O3	−0.2048 (2)	0.71168 (14)	0.79558 (9)	0.0562 (5)	

### Atomic displacement parameters (Å^2^)

**Table:**

	*U*^11^	*U*^22^	*U*^33^	*U*^12^	*U*^13^	*U*^23^
C1	0.0601 (15)	0.0381 (13)	0.0553 (13)	0.0009 (12)	−0.0041 (12)	−0.0057 (10)
C2	0.0419 (12)	0.0429 (12)	0.0412 (11)	0.0069 (10)	−0.0025 (10)	−0.0031 (9)
C3	0.0349 (11)	0.0410 (11)	0.0366 (10)	0.0006 (10)	−0.0014 (9)	−0.0027 (9)
C4	0.0382 (11)	0.0420 (12)	0.0358 (10)	0.0022 (10)	−0.0006 (9)	−0.0008 (9)
C5	0.0424 (13)	0.0459 (13)	0.0433 (12)	−0.0039 (11)	0.0011 (10)	0.0043 (10)
C6	0.0374 (12)	0.0613 (16)	0.0539 (13)	−0.0058 (12)	0.0048 (11)	0.0031 (12)
C7	0.0348 (12)	0.0621 (15)	0.0540 (13)	0.0065 (12)	0.0018 (10)	−0.0024 (12)
C8	0.0421 (12)	0.0444 (12)	0.0410 (11)	0.0045 (11)	−0.0014 (10)	0.0026 (9)
C9	0.0588 (14)	0.0397 (12)	0.0427 (12)	0.0091 (12)	−0.0023 (12)	0.0030 (9)
C10	0.0676 (16)	0.0409 (12)	0.0437 (12)	0.0020 (13)	−0.0013 (12)	0.0066 (10)
C11	0.0595 (16)	0.0503 (14)	0.0541 (14)	−0.0097 (13)	0.0074 (12)	0.0093 (12)
C12	0.103 (2)	0.0414 (14)	0.0578 (15)	−0.0049 (17)	−0.0003 (17)	0.0088 (12)
C13	0.130 (3)	0.0387 (13)	0.0591 (16)	0.016 (2)	0.0005 (19)	0.0022 (12)
C14	0.101 (3)	0.0517 (16)	0.0663 (17)	0.0253 (18)	0.0034 (17)	−0.0017 (13)
C15	0.0702 (17)	0.0507 (14)	0.0608 (15)	0.0181 (14)	0.0031 (14)	0.0013 (12)
C16	0.0371 (12)	0.0480 (13)	0.0666 (14)	−0.0062 (11)	0.0028 (11)	0.0018 (11)
C17	0.0514 (14)	0.0671 (16)	0.0586 (14)	−0.0085 (15)	0.0149 (12)	0.0018 (13)
C18	0.096 (3)	0.151 (4)	0.0661 (19)	0.017 (3)	−0.009 (2)	−0.003 (2)
C19	0.145 (5)	0.254 (8)	0.064 (2)	0.017 (6)	−0.012 (3)	−0.020 (3)
C20	0.203 (7)	0.214 (7)	0.076 (3)	−0.038 (6)	0.041 (4)	−0.055 (4)
C21	0.209 (7)	0.148 (5)	0.100 (3)	0.019 (5)	0.060 (4)	−0.043 (4)
C22	0.124 (3)	0.094 (3)	0.084 (2)	0.021 (3)	0.032 (2)	−0.010 (2)
C23	0.0621 (16)	0.0460 (13)	0.0666 (16)	0.0194 (13)	−0.0007 (14)	−0.0110 (12)
C24	0.0457 (13)	0.0493 (13)	0.0615 (14)	0.0066 (12)	0.0049 (12)	0.0079 (11)
C25	0.119 (3)	0.093 (3)	0.106 (3)	0.061 (3)	−0.024 (3)	−0.004 (2)
C26	0.148 (4)	0.148 (4)	0.133 (4)	0.082 (4)	−0.040 (4)	0.016 (4)
C27	0.092 (3)	0.152 (4)	0.087 (3)	0.008 (3)	−0.027 (2)	0.044 (3)
C28	0.093 (2)	0.111 (3)	0.0569 (17)	−0.014 (2)	−0.0090 (17)	0.0104 (17)
C29	0.0717 (19)	0.0707 (17)	0.0618 (16)	0.0085 (17)	−0.0029 (14)	0.0048 (14)
N1	0.0427 (11)	0.0397 (10)	0.0497 (10)	−0.0022 (9)	0.0009 (9)	−0.0037 (8)
N2	0.0468 (11)	0.0403 (11)	0.0582 (11)	0.0097 (9)	−0.0025 (10)	−0.0058 (9)
O1	0.0786 (14)	0.0369 (9)	0.1073 (17)	−0.0058 (10)	0.0062 (12)	−0.0017 (9)
O2	0.0745 (14)	0.0620 (12)	0.1188 (18)	−0.0200 (12)	0.0288 (14)	0.0139 (12)
O3	0.0412 (9)	0.0526 (10)	0.0749 (12)	0.0058 (8)	−0.0080 (8)	0.0012 (9)

### Geometric parameters (Å, °)

**Table:**

C1—O1	1.209 (3)	C16—C17	1.503 (4)
C1—N2	1.374 (3)	C16—H16A	0.9700
C1—N1	1.397 (3)	C16—H16B	0.9700
C2—C7	1.370 (3)	C17—C22	1.351 (5)
C2—N2	1.377 (3)	C17—C18	1.373 (5)
C2—C3	1.412 (3)	C18—C19	1.362 (6)
C3—N1	1.387 (3)	C18—H18	0.9300
C3—C4	1.398 (3)	C19—C20	1.353 (9)
C4—C5	1.418 (3)	C19—H19	0.9300
C4—C8	1.469 (3)	C20—C21	1.353 (9)
C5—C6	1.383 (3)	C20—H20	0.9300
C5—C11	1.488 (4)	C21—C22	1.375 (7)
C6—C7	1.390 (4)	C21—H21	0.9300
C6—H6	0.9300	C22—H22	0.9300
C7—H7	0.9300	C23—N2	1.460 (3)
C8—O3	1.220 (3)	C23—C24	1.504 (4)
C8—C9	1.486 (3)	C23—H23A	0.9700
C9—C10	1.387 (4)	C23—H23B	0.9700
C9—C15	1.391 (4)	C24—C25	1.354 (4)
C10—C12	1.396 (4)	C24—C29	1.371 (4)
C10—C11	1.483 (4)	C25—C26	1.389 (6)
C11—O2	1.215 (3)	C25—H25	0.9300
C12—C13	1.380 (5)	C26—C27	1.338 (6)
C12—H12	0.9300	C26—H26	0.9300
C13—C14	1.376 (5)	C27—C28	1.344 (5)
C13—H13	0.9300	C27—H27	0.9300
C14—C15	1.382 (4)	C28—C29	1.388 (4)
C14—H14	0.9300	C28—H28	0.9300
C15—H15	0.9300	C29—H29	0.9300
C16—N1	1.467 (3)		
			
O1—C1—N2	126.5 (2)	H16A—C16—H16B	107.9
O1—C1—N1	127.0 (2)	C22—C17—C18	118.3 (3)
N2—C1—N1	106.4 (2)	C22—C17—C16	121.8 (3)
C7—C2—N2	129.7 (2)	C18—C17—C16	119.9 (3)
C7—C2—C3	122.8 (2)	C19—C18—C17	120.2 (5)
N2—C2—C3	107.4 (2)	C19—C18—H18	119.9
N1—C3—C4	133.6 (2)	C17—C18—H18	119.9
N1—C3—C2	106.54 (18)	C20—C19—C18	120.6 (5)
C4—C3—C2	119.8 (2)	C20—C19—H19	119.7
C3—C4—C5	116.6 (2)	C18—C19—H19	119.7
C3—C4—C8	124.2 (2)	C21—C20—C19	120.2 (5)
C5—C4—C8	118.87 (18)	C21—C20—H20	119.9
C6—C5—C4	121.9 (2)	C19—C20—H20	119.9
C6—C5—C11	117.9 (2)	C20—C21—C22	118.9 (6)
C4—C5—C11	120.1 (2)	C20—C21—H21	120.6
C5—C6—C7	121.2 (2)	C22—C21—H21	120.6
C5—C6—H6	119.4	C17—C22—C21	121.8 (5)
C7—C6—H6	119.4	C17—C22—H22	119.1
C2—C7—C6	117.5 (2)	C21—C22—H22	119.1
C2—C7—H7	121.3	N2—C23—C24	113.6 (2)
C6—C7—H7	121.3	N2—C23—H23A	108.8
O3—C8—C4	122.4 (2)	C24—C23—H23A	108.8
O3—C8—C9	120.5 (2)	N2—C23—H23B	108.8
C4—C8—C9	117.0 (2)	C24—C23—H23B	108.8
C10—C9—C15	120.3 (2)	H23A—C23—H23B	107.7
C10—C9—C8	120.5 (2)	C25—C24—C29	118.0 (3)
C15—C9—C8	119.2 (2)	C25—C24—C23	117.6 (3)
C9—C10—C12	119.8 (3)	C29—C24—C23	124.3 (2)
C9—C10—C11	120.4 (2)	C24—C25—C26	120.7 (4)
C12—C10—C11	119.8 (3)	C24—C25—H25	119.7
O2—C11—C10	121.1 (2)	C26—C25—H25	119.7
O2—C11—C5	121.3 (3)	C27—C26—C25	120.9 (4)
C10—C11—C5	117.5 (2)	C27—C26—H26	119.5
C13—C12—C10	119.3 (3)	C25—C26—H26	119.5
C13—C12—H12	120.4	C26—C27—C28	119.2 (4)
C10—C12—H12	120.4	C26—C27—H27	120.4
C14—C13—C12	120.8 (3)	C28—C27—H27	120.4
C14—C13—H13	119.6	C27—C28—C29	120.7 (4)
C12—C13—H13	119.6	C27—C28—H28	119.6
C13—C14—C15	120.5 (3)	C29—C28—H28	119.6
C13—C14—H14	119.8	C24—C29—C28	120.3 (3)
C15—C14—H14	119.8	C24—C29—H29	119.8
C14—C15—C9	119.3 (3)	C28—C29—H29	119.8
C14—C15—H15	120.4	C3—N1—C1	109.50 (19)
C9—C15—H15	120.4	C3—N1—C16	129.55 (19)
N1—C16—C17	112.2 (2)	C1—N1—C16	118.3 (2)
N1—C16—H16A	109.2	C1—N2—C2	110.11 (19)
C17—C16—H16A	109.2	C1—N2—C23	122.9 (2)
N1—C16—H16B	109.2	C2—N2—C23	126.6 (2)
C17—C16—H16B	109.2		

### Hydrogen-bond geometry (Å, °)

**Table:**

*D*—H···*A*	*D*—H	H···*A*	*D*···*A*	*D*—H···*A*
C13—H13···O1^i^	0.93	2.39	3.312 (3)	171.
C23—H23A···O2^ii^	0.97	2.47	3.439 (4)	174.

Symmetry codes: (i) *x*, *y*+1, *z*; (ii) −*x*+1, *y*−1/2, −*z*+3/2.
